# Fatty acid oxidation and carnitine palmitoyltransferase I: emerging therapeutic targets in cancer

**DOI:** 10.1038/cddis.2016.132

**Published:** 2016-05-19

**Authors:** Q Qu, F Zeng, X Liu, Q J Wang, F Deng

**Affiliations:** 1 Department of Cell Biology, School of Basic Medical Sciences, Southern Medical University, Guangzhou, China; 2 Department of Clinical Laboratory, The Fifth Affiliated Hospital, Southern Medical University, Guangzhou, China; 3 Department of Pharmacology and Chemical Biology, University of Pittsburgh School of Medicine, Pittsburgh, PA, USA

## Abstract

Tumor cells exhibit unique metabolic adaptations that are increasingly viewed as potential targets for novel and specific cancer therapies. Among these targets, the carnitine palmitoyltransferase system is responsible for delivering the long-chain fatty acid (FA) from cytoplasm into mitochondria for oxidation, where carnitine palmitoyltransferase I (CPTI) catalyzes the rate-limiting step of fatty acid oxidation (FAO). With increasing understanding of the crucial role had by fatty acid oxidation in cancer, CPTI has received renewed attention as a pivotal mediator in cancer metabolic mechanism. CPTI activates FAO and fuels cancer growth via ATP and NADPH production, constituting an essential part of cancer metabolism adaptation. Moreover, CPTI also functionally intertwines with other key pathways and factors to regulate gene expression and apoptosis of cancer cell. Here, we summarize recent findings and update the current understanding of FAO and CPTI in cancer and provide theoretical basis for this enzyme as an emerging potential molecular target in cancer therapeutic intervention.

## FACT

- FAO has the capacity to act as the great power to fuel tumor growth in conditions of metabolic stress.
- CPTI, which catalyzes the rate-limiting step of FAO, is overexpressed in numerous tumors. Inhibition of CPTI is proved to suppress cancer growth.
- Besides FAO, CPTI also functionally intertwines with other key pathways and factors in the regulation of gene expression and apoptosis of cancer cell.
- In tumor microenvironment, CPTI also exerts important properties in tumor neovascularization.

## Open Questions

- What is the specific mechanism of FAO in contributing to cancer survival?
- How do FAO, aerobic glycolysis and fatty acid synthesis (FAS) interact with each other in cancer cells under metabolic stress?
- Are there any other molecules that regulate FAO and CPTI activity or are regulated by CPTI in tumor cells?
- Is it feasible that we explore new anticancer therapies targeting CPTI while minimizing side effects of CPTI inhibition?
- In endothelial cells, will CPTI become a new important therapeutic target for tumor neovascularization?

Altered energy metabolism constitutes the major part of tumor metabolic adaptation and has been well established as a hallmark of cancer.^1, 2^ The best-known metabolic abnormality in cancer cells is the Warburg effect, which is the increased glycolysis in the presence of oxygen.^3^ Apart from alteration in glucose metabolism, there are compelling evidences showing that cancer cells have specific alterations in different aspects of lipid metabolism. These alterations can affect the availability of membrane structural lipids, the synthesis and degradation of lipids that contribute to energy homeostasis and the abundance of lipids with signaling functions.^4^

Recent research has pointed to the crucial role of fatty acid oxidation (FAO) as an essential source of NADH, FADH2, NADPH and ATP, all providing survival advantage to cancer.^5, 6, 7^ As the key rate-limiting enzyme of FAO, carnitine palmitoyltransferase I (CPTI) controls FAO directly and thus facilitates cancer metabolic adaptation. Meanwhile, CPTI also shares multiple connections with many other cellular signaling pathways, making it a multifunctional mediator in cancer pathogenesis.

In this review, we will summarize briefly the biological characteristics of FAO and its key enzyme CPTI. Emphasis will be laid on the confirmed functions of CPTI in various carcinomas and its related pathways in cancer metabolic homeostasis. In the end, we will show some novel findings about the functions of CPTI and further discuss the prospect as well as the problems encountered in targeting CPTI for cancer therapy.

## CPTI Enzymes and FAO

In normal untransformed cells, the balance between fatty FAS (fatty acid synthesis) and FAO (β-oxidation) depends upon nutritional state and tissue mitochondrial metabolism.

**Figure 1:**
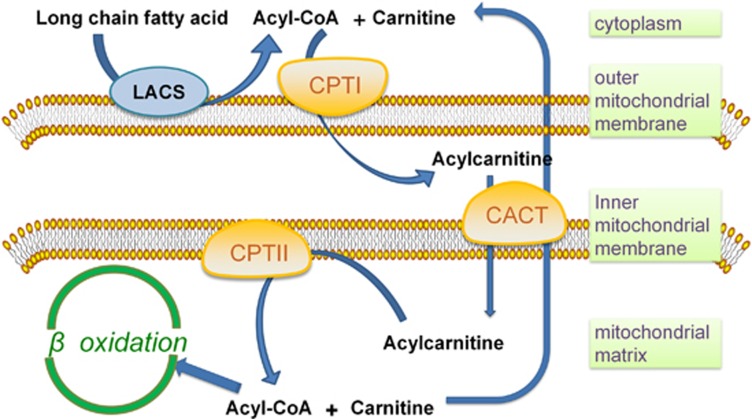
The regulation of FAO on the mitochondrial membrane. Long-chain fatty acid is transformed into acyl-CoA after the catalysis of long-chain acyl-CoA synthetase (LACS). The carnitine palmitoyltransferase system then transport acyl-CoA from cytoplasm into mitochondrial matrix for oxidation: CPTI converts acyl-CoAs into acylcarnitines. CACT exchanges acylcarnitine and carnitine between outer and inner membranes of mitochondrial and finally acylcarnitine is converted back into acyl-CoAs for oxidation by CPTII

FAO mainly occurs in mitochondria and involves a cyclical series of reactions that result in the shortening of fatty acids (two carbons per cycle). These reactions generate NADH, FADH2 and acetyl coenzyme A (CoA) in each round, until the last cycle when two acetyl-CoA molecules are produced from the catabolism of a four-carbon fatty acid. NADH and FADH2 that are generated by FAO enter the electron transport chain to produce ATP.^5^ The first step of FAO is fatty acid activation, producing long-chain acyl-CoA catalyzed by the long-chain acyl-CoA synthetase, which is the prerequisite of long-chain fatty acid catabolism.^8^ There are 26 genes encoding acyl-CoA synthetase that have discriminatory affinities for activating short-, medium-, long- and very long-chain fatty acids, respectively.^9^ Due to the lack of permeability of long-chain acly-CoAs to penetrate the mitochondrial inner membrane, the carnitine palmitoyltransferase system is responsible for transporting long-chain acly-CoAs into mitochondria from cytoplasm. Three components are involved in this transporting system: CPTI, the carnitine acylcarnitine translocase (CACT) and CPTII. CPTI grapples on the mitochondrial outer membrane with its C terminus and N terminus facing the cytoplasm.^10^ It catalyzes the rate-limiting step of FAO by converting acyl-CoAs into acylcarnitines. CACT is an inner membrane protein that exchange acylcarnitine and carnitine between outer and inner mitochondrial membranes. CPTII is located in the matrix side of the mitochondrial inner membrane^11^ and it is responsible for converting acylcarnitine back into acyl-CoAs for oxidation (Figure 1).

The CPTI family of proteins, by shuttling long-chain fatty acid into mitochondria, constitutes the rate-limiting step of FAO.^12^ It comprises of three subtypes, CPTIA, CPTIB and CPTIC, which show tissue-specific distribution.^13, 14^ CPTIA and CPTIB distribute widely in human body and demonstrate considerable similarities; both of them have a central role in mitochondrial β-oxidation. Malonyl-CoA, the product of the first committed step in FAS and usually derived from glucose, is the physiological inhibitor of CPTIA and CPTIB and turns them into enzymes exhibiting strong flux control on FAO.^15^ Although there exists considerable sequence similarity, the sensitivity of these two enzymes to their inhibitor malonyl-CoA differs greatly (CPTIA has a tenfold higher Ki for malonyl-CoA),^15^ which makes CPTIA the more prevailing enzyme that accomplishes the rate-limiting step in *β*-oxidation.^16^

CPTIC is one of the CPTI isoforms expressed exclusively in the brain.^17^ Like CPTIA and CPTIB, the brain-specific CPTIC displays high-affinity binding to malonyl-CoA, but its enzymatic activity cannot be observed using conventional substrates.^18, 19, 20^ Gene-targeting studies have demonstrated that CPTIC was not essential for the survival of mice but these animals did exhibit reduced FAO.^21^ Recent studies showed that CPTIC was localized both in endoplasmic reticulum and mitochondria, but its presence in endoplasmic reticulum is predominant. Despite these progresses, the exact subcellular localization of CPTIC and its cellular function remains unclear.^20^

In summary, although CPTIA, CPTIB and CPTIC have specific tissue distribution, targeted inhibition/depletion of the three isoforms leads to a considerable suppressed phenotype in cancer cells including repressed proliferation, chemoresistance and neovascularization (studies focusing on their functions are listed in Table 1). A deeper understanding of the three isoforms is without doubt needed for developing drugs or therapies targeting these enzymes.

## FAO in Cancer Metabolism

Although the relevance of FAO in cancer cells has not been examined thoroughly like glycolysis, glutaminolysis and FAS, current work has started to bring to light the function of FAO in tumor cells.

### Increasing ATP supply for tumor survival

FAO has the capacity to act as the great power to fuel tumor growth by increasing ATP production in conditions of metabolic stress. For example, CPTIC is upregulated in tumors of the lung, and enhances tumor survival in conditions of metabolic stress (glucose or oxygen deprivation) *in vitro.*^22^ Overexpression of CPTIC in cancer cells promotes FAO and ATP production, adaptation to metabolic stress and resistance to mTOR complex 1 (mTORC1) inhibitors.^18, 23^ Moreover, cells derived from solid tumors that undergo loss of attachment (LOA) to the extracellular matrix (known as anoikis) display inhibition of glucose uptake and catabolism, which results in the loss of ATP, NADPH (as a result of decreased flux through the pentose phosphate pathway) and increased production of reactive oxygen species (ROS).^5^ Interestingly, antioxidants counteract ROS accumulation and reactivate FAO, increase ATP levels and prevent LOA-induced anoikis, although the exact mechanism by which the increasing ATP rescues anoikis remains unclear.^24^

In addition, some tumors such as prostate cancer^25^ and diffuse large B-cell lymphoma, are highly dependent on FAO for survival and growth.^26, 27^ Either genetically or pharmacologically inhibition of FAO reduces ATP supply and impairs cell proliferation in PC3 prostate cancer,^25^ Burkitt's lymphoma^28^ and human glioblastoma.^29^ All evidence showed that FAO contributes to cancer cell proliferation by increasing ATP supply.

### FAO contribution to the total NADPH pool

Apart from producing NADH and FADH2 directly to supply extra ATP for cancer survival,^5^ FAO is also an important source of NADPH. The major effect of FAO in NADPH production lies in its promoting acetyl-CoA generation. The acetyl-CoA enters the Krebs cycle and together with oxaloacetate give rise to citrate, which can be exported to the cytoplasm and engage in NADPH-producing reactions: the transformation of malate to pyruvate catalyzed by malic enzyme (ME1) and the oxidation of isocitrate into α-ketoglutarate catalyzed by isocitrate dehydrogenase (IDH1).^5^

The production of FAO-derived cytosolic NADPH provides redox power for cancer cells to counteract oxidative stress. For example, inhibition of FAO in human glioblastoma cells by etomoxir, a CPTI inhibitor, impairs NADPH production and increases ROS, resulting in ATP depletion and cell death.^30^ Next, NADPH is a coenzyme for anabolic enzymes in the synthesis of fatty acids and cholesterol, and is thus the key for the generation of new building blocks that sustain cell growth and proliferation.^31^ Current data further showed that the function of FAO in maintaining NADPH homeostasis is regulated through the LKB1-AMPK axis.^32^ When starved from glucose, cells lacking the ability to activate AMPK show NADPH depletion, increased oxidative stress and increased apoptosis.^33^

### FAO in tumor chemoresistance and hypoxia

Chemoresistance is one of the essential ways that cancer evades from death and a major cause of treatment failure in patients with cancer. As is mentioned above, CPTIC overexpression in cancer cells promotes FAO and resistance to mTORC1 inhibitors. In contrast, the inhibition of FAO sensitizes cancer cells to drugs and helps cancer to overcome chemoresistance. For example, the inhibition of FAO inhibited paclitaxel-resistant lung adenocarcinoma cell proliferation^34^ and increased the resistance of chronic lymphocytic leukemia to glucocorticoids.^35^ These studies suggested that FAO inhibition is a potential strategy to overcome drug resistance in tumors.

Besides chemoresistance, the ability to survive in hypoxia is also significant for tumor cells. How do FAO work under hypoxia in cancer cells? In tumor tissue, intermittent blood supply leads to an alternating situation of lack-of-oxygen and reoxygenation, which causes oxidative stress.^23, 36^ Hypoxia induces a HIF-1*α*-dependent accumulation of lipid droplets and decreased FAO in tumor cells, whereas the hypoxia-reoxygenation leads to increased FAO in MCF-7 breast cancer cells, thereby providing reduced power to protect against accumulation of ROS.^37^ Current studies further demonstrated that HIF-1 inhibited two enzymes in FAO, the medium- and long-chain acyl-CoA dehydrogenases MCAD and LCAD. And LCAD loss further promotes cancer by reduction of PTEN, highlighting the significance of FAO inhibition in cancer.^38^ However, in lung cancer and breast cancer, tumor cells constitutively expressing CPTIC show increased FAO, ATP production and resistance to hypoxia.^18^ Further study shows that CPTIC is induced by AMPK activation under hypoxia and a p53–AMPK–CPTIC axis evoked in response to metabolic stress has also been implicated.^18, 39^

### FAO and aerobic glycolysis

**Figure 2:**
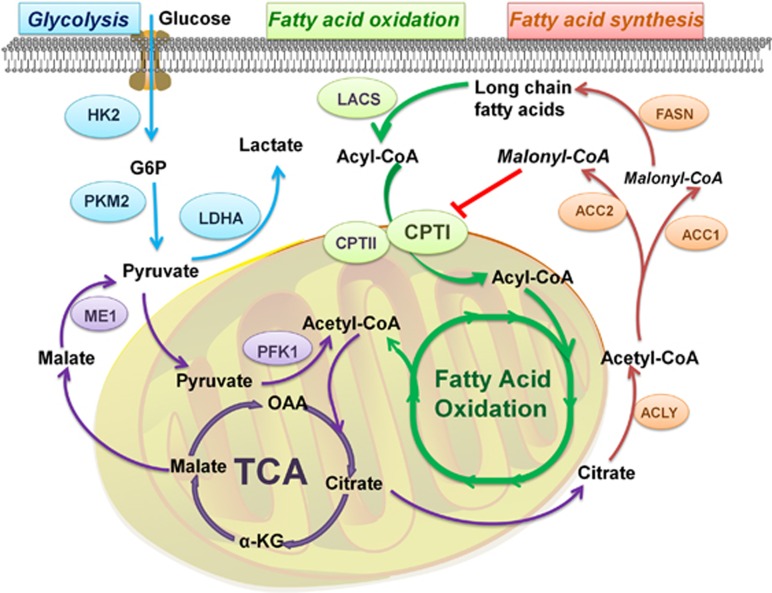
Metabolic pathway of aerobic glycolysis, FAS and FAO. CPTI can be inhibited directly by ACC2-generated malony-CoA, a crucial intermediate in FAS. This effect prevents FAS and FAO from being activated simultaneously. FAO also takes the long-chain fatty acids as raw materials, which are the products of FAS. FAO and aerobic glycolysis are both significant energy-supplying processes in cancer. Acetyl-CoA generated from FAO serves as an essential source of tricarboxylic acid cycle (TCA), which can finally produce malate to supplement pyruvate

Aerobic glycolysis is perceived as the dominant energy-supplying pathway to fulfill the high demand of the rapid proliferating tumor cells.^40^ However, emerging evidence is indicating that some tumors, including prostate tumor, leukemia and large B-cell lymphoma, utilize FAO as their main energy supply for proliferation and survival.^26, 41, 42^ For example, prostate cancer has low avidity for FDG (2-deoxy-2-fluoro-d-glucose) due to enhanced FAO and decreased glycolysis, therefore cannot get ideal FDG-PET imaging result.^43^ Furthermore, inhibition of CPTI by etomoxir leads to activated aerobic glycolysis and potentiated FDG signaling.^44^ It is reasonable to speculate that the inhibition of CPTI leads to the activation of aerobic glycolysis, which elicits complementary effects for energy loss due to impaired FAO. More in-depth analysis is required to determine whether the complementary effect results from the direct interaction between FAO and aerobic glycolysis, or is mediated by other enzymes responded to the energy deprivation (such as AMPK; Figure 2).

### FAO and FAS

*De novo* FAS is amplified in many cancers and is believed to be used mainly for the newly synthesized membrane phospholipids as well as the signal molecules, which are needed for the cancer cells' rapid proliferation.^45^ Also, FAO is indispensible for many cancers especially under metabolic stress. FAO and FAS are antagonistic and incompatible in relationship.^16, 46, 47^ Moreover, current data further demonstrated that genetic manipulation of acetyl-CoA carboxylase 1 (ACC1) or acetyl-CoA carboxylase 2 (ACC2) in cancer cells yielded different outcomes in terms of FAS and FAO.^5^ ACC2-generated malonyl-CoA can inhibit CPTI and thus control FAO, but ACC1-derived malonyl-CoA is just an intermediate for FAS and exerts no suppressive effect on CPTI.^48, 49^ Therefore, ACC determines which pathway is active, depending on the level of acetyl-CoA and malonyl-CoA.^5^ In addition, simultaneous suppression of FAS and FAO has been demonstrated to be quite effective in anticancer therapy in some cancers (e.g., prostate cancer, myeloma).^50, 51^ All results above show that FAS and FAO may support each other.^26^

Although still under debate, there are two reasonable explanations for the simultaneous suppression of FAS and FAO. Firstly, different effects of the compartmentalized malony-CoA on CPTI may contribute to the co-activation. Secondly, the free fatty acid needed for FAO not only comes from extracellular environment but also originates from de novo lipogenesis. FAS supplies the material for FAO, and meanwhile FAO promotes the accumulation of acetyl-CoA that is needed to initiate FAS^26^ (Figure 2).

## CPTI Promotes Cancer Survival Under Metabolic Stress

Numerous studies have shown that the overexpression of CPTI is tightly associated with tumor progression in breast cancer,^52, 53, 54^ gastric cancer,^55^ prostate cancer,^25, 50^ lung cancer,^18^ ovarian cancer,^56, 57^ hepatoma,^58^ myeloma^51^ and high grade glioblastoma^29, 59^(roles of CPTI in various tumors are summarized in Table 1).

CPTI serves to activate FAO that increases ATP and NADPH reserves, protecting cancer against the environmental stress such as glucose deprivation and hypoxia.^60, 61^ CTP1C expression displays an inverse correlation with the activation of mTOR pathway and attenuates tumor sensitivity to rapamycin (a mTOR inhibitor),^18^ which suggests that the CPTIC-involved pathway parallels to mTOR-enhanced glycolysis.^62^ Moreover, CPTIC is an essential downstream mediator of the AMPK pathway as well as a target gene of p53 in a p53/AMPK-dependent manner.^39^

Knockdown or inhibition of CPTI by inhibitors (such as *etomoxir* and *ST1326*) suppresses cancer cell growth. CPTI is proved to have the potential to be a new target in anticancer treatment (e.g., breast cancer,^54^ prostate cancer,^50^ leukemia^7, 63^). For example, inhibition of CPTIA results in impaired cancer cell proliferation in acute myeloid leukemia.^7, 28, 64^ Pharmacological inhibition of CPTIA by *ST1326* results in intensive cytotoxicity in Burkitt's lymphoma. Interestingly, altered β-oxidation followed by inhibition of CPTIA remarkably attenuate c-myc–mediated lymphomagenesis, implying a potential role CPTI had in the pathogenesis of c-myc–driven cancer.^28^

## Essential Role of CPTI in Cancer Cell Apoptosis

### CPTI and bcl-2 family

**Figure 3:**
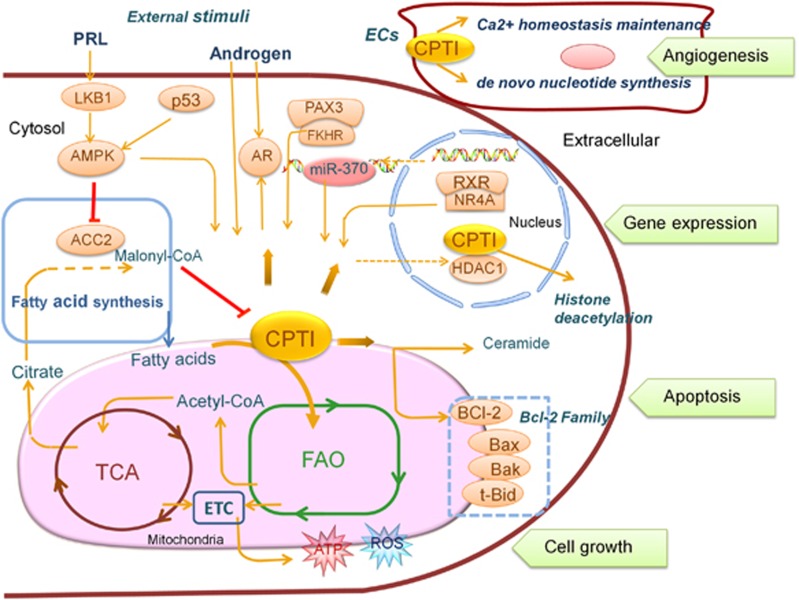
CPTI catalyzes the rate-limiting step of FAO and directly alters its intensity, supplying more ATP and ROS to facilitate cancer cell growth. CPTI also associates with other key regulatory pathways and factors such as aerobic glycolysis and FAS, p53/AMPK axis, PAX3-FKHR and external stimuli such as hormone (e.g., PRL, androgen). CPTI modulates apoptosis of cancer cells via interaction with BCL-2 family and cytotoxic lipids (e.g., ceramide). MiR-370 can regulate the expression of CPTI. The relations of CPTI with various nuclear proteins provided clues to its function in gene expression: transcriptional regulation by RXR/NR4A and deacetylation regulation by recruiting HDAC complexes. In the endothelial cells (ECs) outside the cancer cells, the high expression of CPTI can influence tumor neovascularization enormously

Recent studies demonstrated that the truncated Bid of the bcl-2 family decreased CPT-1 activity in a malony-CoA-independent manner, thereby resulting in the accumulation of palmitoyl-coenzyme A (CoA) and cancer cell apoptosis.^65^ And the overexpression of Bcl-2 cripples this effect through direct interaction with CPTIA.^66^ In contrast, pharmacologic inhibition of FAO with etomoxir or ranolazine inhibited cancer cell proliferation and sensitized human leukemia cells to apoptosis induced by ABT-737, a molecule that released proapoptotic Bcl-2 proteins such as Bak from antiapoptotic family members, suggesting that CTP1 can antagonize Bcl-2-induced apoptosis. Mechanistically, after ABT-737 treatment, the inhibition of FAO facilitates the formation of Bak-dependent mitochondrial permeability transition independently of p53 (ref. 42; Figure 3).

### CPTI and cytotoxic lipids

An alternative way through which CPTI regulates tumor apoptosis is by controlling the production of potentially toxic lipid metabolites such as ceramide and its precursors—palmitoyl-CoA and palmitic acid. In prostate cancer cells, inhibition of CPTIA by *etomoxir* increases substantially the level of ^16^C and ^18^C containing-ceramides,^50^ which are synthesized *de novo* by palmitic acid and stearic acid and exerts the most dramatic impact on intrinsic apoptosis activity.^67^ Moreover, the palmitoyl-CoA and palmitate of ceramide precursors also induce apoptosis via *de novo* synthesis of ceramide.^66, 68^ CPTIA can protect cells from apoptosis by clearing the cytoplasmic long-chain fatty acyl-CoA such as palmitoyl-CoA and thus impede the production of ‘palmitate/palmitoyl-CoA/ceramide'.

## Regulation of CPTI Expression in Cancer Cells

### MiR-370 regulates CPTIA in hepatocellular carcinoma cells

MicroRNAs are small noncoding RNAs which serve as essential endogenous regulators for gene expression at the post-transcriptional level.^69, 70^ Certain sets of circulating microRNAs might function as a tumor surveillance mechanism exerting continuous inhibition on tumor formation.^71, 72^ In hepatocellular carcinoma (HepG2), CPTIA is downregulated by miR-370 which targets the 3′ untranslated region of CPTIA.^73^ Bio-information and molecular analysis demonstrated that CPTIA is a direct target of miR-370 and the rate of FAO reduced by 40% due to the downregulation of CPTIA by miR-370. Meanwhile, the lipogenic genes SREBP-1c, DGAT2, FAS and ACC1 can be activated by the overexpression of the direct lipogenesis regulator miR-122 through the effect of miR-370. It was hypothesized that in HepG2, miR-370 contributed to hepatic triglyceride accumulation by directly downregulating CPTIA and promoting lipogenesis through the activation of miR-122.^73^ Therefore, miR-370 is regard as a potential regulator of CPTI gene expression.

### CPTI serves as a downstream effector of PAX3-FKHR in ARMS

CPTI expression is regulated by a different pathway in aggressive alveolar rhabdomyosarcoma (ARMS). It has been confirmed that the downregulation of CPTIA decreases the motility of human ARMS cell line Rh30, and CPTIA also serves as a transcriptional target of a PAX3-FKHR fusion transcription factor, which regulates cell migration and promotes metastasis.^74^ As a fusion transcription factor produced by chromosomal translocation, PAX3-FKHR has been linked closely to cell differentiation, metastasis, and migration of ARMS.^75, 76, 77^ This is the first study showing that CPTIA contributes to cancer cell motility as the downstream effector of PAX3-FKHR, implying a potential value of targeting CPTIA for ARMS therapy^74^ (Figure 3).

### Transcriptional regulation of CPTIA by RXR-NR4A receptor

Retinoid X receptors (RXRs) are members of the nuclear receptor superfamily and can be activated by 9-cis retinoic acid. RXRs form homodimers and heterodimers with other nuclear receptors such as the retinoic acid receptor and NR4 subfamily nuclear receptors, Nur77 and NURR1. RXR has a vital role in the transcription of tumor suppressor genes which induce differentiation and suppress cancer growth.^78, 79^ As NR4 subfamily nuclear receptors (e.g., Nur77, NURR1) are ubiquitous heterodimerization partners of RXR in forming heterodimers,^80^ current study also revealed that when the cancer cells are incubated with HX600, a selective RXR agonist for Nur77-RXR and NURR1-RXR, the expression of CPTIA is induced through Nur77 or NURR1-mediated mechanism in the teratocarcinoma cells or heptoma carcinoma HepG2 cells^81^ (Figure 3).

### Regulation of CPTI by hormone in tumor cells

A large body of literature supports that prolactin (PRL) released from the anterior pituitary gland promotes cell proliferation, survival, migration/invasion and angiogenesis.^82^ In MCF-7 and MDA-MB-231 breast cancer cells, recent study showed that PRL stimulation increased the expression of CPTIA (liver isoform) at both mRNA and protein level, and PRL-mediated effects are partially dependent on the LKB1-AMPK pathway.^53^ This result suggests that PRL may enhance fatty acid β-oxidation by stimulating CPTI expression and/or activity in breast cancer cells (Figure 3).

Androgen increases the production of ROS in prostate cancer cells, which has been postulated to have a key role in the initiation and progression of prostate cancer.^83^ Furthermore, R1881 (synthetic androgen) increased the expression and activity of CPTI, the rate-limiting enzyme in the process of mitochondrial FAO, through AR-mediated pathway, leading to increased production of ROS associated with prostate cell proliferation and mutagenesis.^84^ On the other hand, decreased expression of AR isoform in prostate cancer is also detected after the inhibition of CPTIA by *etomoxir*.^50^ These results not only underscore the importance of CPTIA in modulating AR in prostate cancer,^50^ but also indicate that there exist mutual feedback regulations between AR and CPTI (Figure 3).

### Regulation of CPTI by histone deacetylation

Histone deacetylase 1 (HDAC1) has a crucial role in transcriptional repression of gene expression.^85, 86^ Current data showed that the nuclear localization of CPTI existed exclusively in neoplastic cells, and HDAC1 and CPTI coimmunoprecipitats in nuclear extracts from MCF-7 cells. The treatment with HDAC inhibitors such as trichostatin A and butyrate significantly decreased nuclear expression of CPTI and its bond to HDAC1^86^ (Figure 3). The peculiar localization of CPTI in the nuclei of human carcinomas and the functional link between nuclear CPTI and HDAC1 suggest that CPTI may act as a new role in the histonic acetylation level of tumor, but the function and mechanism need to be further explored.

## The Therapeutic Window of CPTI in Cancer Therapy

The inhibition of CPTI irreversibly suppressed FAO, which was thought to be a potential anticancer target in many studies. The inhibition drugs of CPTI have already been developed in the treatment of heart diseases. Among which the inhibitory effect of etomoxir on CPTI is fully demonstrated.^87^ As an irreversible inhibitor of CPTI, etomoxir has been developed as a drug for treating type 2 diabetes mellitus and chronic heart failure for a long time.^88, 89, 90^ Recently, etomoxir has gained decent anticancer results in many experiments. Nevertheless, etomoxir is proved to have some serious toxic effect^91^ and thus is still in the preclinical stage.^5^ Previous studies presented that etomoxir treatment induced cardiac hypertrophy by increasing oxidative stress and stimulating NF-κB.^91, 92^ What is more, the suppression of CPTI by etomoxir eventually reactivated the original low level glycolysis in prostate cancer.^44^ Other CPTI inhibitory drugs, such as perhexiline, is also proved to be toxic (such as neurotoxicity and hepatotoxicity) especially after long-term therapy.^93^ These side effects are caused mainly by the low selectivity of the drugs—inhibiting CPTI in target spot as well as in normal ones, leading to metabolic disorder. Based on such background, a more selective CPTI inhibitor named ST1326 (Teglicar) is developed.^94^ Its effect is reversible and liver specific, showing impressive safety records except for the fat liver, as expected.^95^ ST1326 is now in phase 2 studies for the treatment of type 2 diabetes and is also showing great potential in the treatment of leukemias.^96, 97^

## Concluding Remarks

The aberrant expression of CPTI directly alters the intensity of FAO, acclimatizing cancer cells to metabolic stress. In spite of FAO, there are other key regulatory pathways through which CPTI modulates growth, gene expression and apoptosis of cancer cells. Depending on cancer types, the function of CPTI couples with diverse modulators in specific cancer and can be modulated at different levels. CPTI is also indispensable at the junction of FAO, aerobic glycolysis and FAS. There may exist undiscovered ‘bridges' involving CPTIA that coordinate the three biological processes, which is crucial to the metabolic adaption in cancer.

In cancer cells, CPTI acts as a multifunctional enzyme and benefits cancer survival as well as growth at multiple fronts. In tumor microenvironment, CPTI that expresses in non-tumorous cells also has a major impact on cancer survival. In endothelial cells (ECs), overexpressed CPTI can promote cancer growth through acceleration of tumor neovascularization.^98^ A recent article on *Nature* indicates that loss of CPTIA in the ECs results in impaired cancer cell proliferation and inhibits *de novo* nucleotide synthesis and DNA replication.^98^ In another study, it has been shown that the inhibition of CPTIA in ECs induces hyperpermeability *in vitro* and leakage of blood vessel *in vivo* through increasing Ca^2+^ oscillation frequency.^99^ These novel studies point to an important role that CPTI had in promoting cancer angiogenesis. In the future, further efforts should be directed to interpret the precise molecular mechanism of CPTI in cancer angiogenesis and fully tap the potential of CPTI inhibition in cancer therapeutic intervention.

## Figures and Tables

**Table 1:** Overview of CPTI roles in cancer biology

**CPTI isoform**	**Cancer type**	**Significance**	**Reference**
CPTIA	Prostate cancer	Upregulated expression; its inhibition has antitumor actions	25, 41, 50
	Burkitt's lymphoma	Upregulated expression	28
	Glioblastoma	Upregulated expression	29, 30
	Lymphocytic leukemia	Its inhibition has antitumor actions; contributes to cancer chemoresistance	7, 35, 42
	Breast cancer	Upregulated expression; its inhibition has antitumor actions; acts as a new role in the histonic acetylation level of tumor	37, 52, 53, 54, 55, 86
	Gastric cancer	Oleic acid activates its expression	55
	Ovarian cancer	Upregulated expression	56, 57
	Alveolar rhabdomyosarcoma	Contributes to cancer cell motility	74
	Teratocarcinoma	Transcriptional regulation of it by RXR-NR4A receptor	81
	Endothelial cells	Upregulated expression; contributes to tumor neovascularization	98, 99
CPTIC	Lung cancer	Upregulated expression contributes to cancer chemoresistance	18
	Colon	Its inhibition has antitumor actions	18
	Breast	Upregulated expression	18, 62
	Glioblastoma	Upregulated expression	29, 59
CPTI (unspecified isoform)	Hepatoma	Upregulated expression	58, 60
	Acute myeloid leukemia	Its inhibition has antitumor actions	63
	Diffuse large B-cell lymphoma	Upregulated expression	26, 27
	Myeloma	Its inhibition has antitumor actions	51
	Lung cancer	Contributes to cancer chemoresistance	34

Abbreviation: CPTI, carnitine palmitoyltransferase I

## Abbreviations

FA: fatty acid
CPTI: carnitine palmitoyltransferase I
FAO: fatty acid oxidation
FAS: fatty acid synthesis
CACT: carnitine acylcarnitine translocase
mTORC1: mTOR complex 1
LOA: loss of attachment
ROS: reactive oxygen species
FDG: 2-deoxy-2-fluoro-D-glucose
ACC1: acetyl-CoA carboxylase 1
ACC2: acetyl-CoA carboxylase 2
CoA: coenzyme A
ARMS: alveolar rhabdomyosarcoma
RXRs: retinoid X receptors
PRL: prolactin
HDAC1: histone deacetylase 1
ECs: endothelial cells
